# Psychological Impact of Health Risk Communication and Social Media on College Students During the COVID-19 Pandemic: Cross-Sectional Study

**DOI:** 10.2196/20656

**Published:** 2020-11-18

**Authors:** Mengyao Li, Li Liu, Yilong Yang, Yang Wang, Xiaoshi Yang, Hui Wu

**Affiliations:** 1 China Medical University Shenyang China

**Keywords:** COVID-19, anxiety, panic, health risk, communication, social media

## Abstract

**Background:**

The outbreak of COVID-19 began in 2019 and is expected to impact the psychological health of college students. Few studies have investigated the associations among health risk communication, social media, and psychological symptoms during a major pandemic.

**Objective:**

The aim of this research was to assess the prevalence of psychological symptoms among college students and explore their associations with health risk communication and social media.

**Methods:**

A web-based survey was distributed through the Wenjuanxing platform among Chinese college students from March 3-15, 2020. In addition to demographics, information on health risk communication and social media was collected, and the Symptom Checklist 90 Phobia and Health Anxiety Inventory subscale was used to assess psychological symptoms among 1676 college students in China. Multivariable logistic regression was performed to examine these independent risk factors.

**Results:**

The prevalence of panic and health anxiety was 17.2% (288/1676) and 24.3% (408/1676), respectively. Regarding risk communication, understanding the risk of COVID-19 (odds ratio [OR] 0.480, 95% CI 0.367-0.627) was a protective factor against panic. Knowledge of prognosis (OR 0.708, 95% CI 0.551-0.910), preventive measures (OR 0.380, 95% CI 0.195-0.742), and wearing face masks (OR 0.445, 95% CI 0.230-0.862) were shown to be protective factors in predicting health anxiety. Perceived lethality (OR 1.860, 95% CI 1.408-2.459), being affected by the global spread (OR 1.936, 95% CI 1.405-2.669), and impact on social contacts (OR 1.420, 95% CI 1.118-1.802) were identified as significant risk factors associated with health anxiety. In terms of social media, trust in mainstream media (OR 0.613, 95% CI 0.461-0.816) was considered to be a protective factor against health anxiety.

**Conclusions:**

There was a high prevalence of psychological symptoms among college students. Health risk communication and social media use were important in predicting psychological symptoms, especially health anxiety. Scientific and evidence-based information should be reported by social media platforms. Web-based consultation and intervention measures should be the focus of future studies.

## Introduction

The COVID-19 pandemic is a global health threat [1]. The World Health Organization (WHO) has declared the COVID-19 outbreak to be a public health emergency of international concern [2]. The COVID-19 pandemic has spread worldwide and has had a significant impact on public health, governments, and social systems [3]. As of April 26, 2020, the number of global cumulative confirmed cases had exceeded 2,800,000, and the cumulative death toll was 192,971 [4].

The pandemic is not only leading to rapidly increasing numbers of confirmed cases and deaths but is also having a psychological impact on patients and health care workers who are exposed to COVID-19 [5]. Although most people are free from COVID-19 infection, they are being psychologically impacted by the COVID-19 pandemic [6,7]. People who are quarantined may have numerous psychological symptoms, such as anxiety, depression, panic, guilt, and stress [8]. Panic and anxiety are two common psychological symptoms experienced during the outbreak; this is likely related to the limited knowledge about COVID-19 and its highly infectious nature [8-11]. These psychological symptoms may cause adverse events and further contribute to social instability and racism [12].

College students are a key population of concern during the COVID-19 pandemic. The college years are a peak period for psychological symptom onset [13]. The high prevalence of COVID-19, home quarantine measures, shortage of masks during the initial stage of the pandemic, and delays in opening schools, colleges, and universities are expected to aggravate the psychological symptoms of college students. The mental health of college students is significantly affected during public health emergencies [14]. The WHO indicated that almost 90% of the world`s students (more than 1,500,000,000 children and young people) have been affected by nationwide school closures [15]. It is necessary to help college students maintain and develop their psychological health during the pandemic.

Researchers have increasingly acknowledged the importance of consistent, clear, and effective health risk communication throughout a pandemic [16]. Risk communication refers to an interactive process of exchanging information among individuals, groups, and institutions [17]. Health risk communication has a critical impact on the spread of pandemics and may help public health officials improve pandemic strategies and messaging [18]. Effective risk communication contributes to the implementation of precautionary behaviors, especially when new pandemic infectious disease risks arise in the phase when no treatment is available [19]. Based on protection motivation theory [20] and knowledge-attitude-practice theory [21], risk perception, correct knowledge, understanding, attitudes, and skills [16,22,23] are key to promoting and implementing preventive behavior [19,24]. Health risk communication is also important in maintaining psychological health. Emotional experiences during a hazardous event can influence individuals’ evaluation of the negative outcomes of that event [25]. Previous studies have found that health risk communication can be a predictor of postdisaster mental health [26,27]. Risk perception is associated with mental symptoms, and dread of risk can increase the risk of mental symptoms [28]. Risk perception is associated with psychological health in earthquake survivors [29]. However, the association between health risk communication and psychological health during a pandemic has not yet been established, especially among college students.

Mass media plays a critical role during new and rapidly spreading global health risks [30]. In China, official departments provide daily updates about surveillance and active cases through social media [10]. This information helps the public improve their awareness of the outbreak. In addition, in a study by Gao et al [31], 82% of participants were found to frequently engage with social media. In addition to mainstream media, websites release and transfer COVID-19–related information [10]. The proliferation of internet-based health news encourages the referencing of media and academic articles, which may exaggerate the strength of results and mislead the public [32]. Contradictory, doubtful (untrustworthy), false, or misleading information may lead to public panic and, in turn, cause mental health symptoms [33]. These untrusted media sources heighten confusion and fearmongering [8,32] and cause anxiety and panic among students. As young people, college students are willing to obtain information on the internet. Therefore, college students may access more information sources and more complex content and thus may be more seriously affected [34,35]. This phenomenon may have a significant psychological impact on college students.

Thus, the COVID-19 pandemic may place a great psychological burden on college students worldwide. Health risk communication and trusted sources of information regarding COVID-19 are essential to prevent excessive panic and anxiety and to manage the outbreak in a scientific and effective way. The aims of this study are to explore the prevalence of panic and anxiety among college students and to identify the relationships among health risk communication, social media, and these two psychological symptoms. By identifying factors associated with negative psychological impacts, we hope that future research and interventions can be developed from our findings.

## Methods

### Ethics Statement

The procedures used in the current study were approved by the Committee on Human Experimentation of China Medical University (YDJK2020022). All subjects voluntarily enrolled in this research. Informed consent was provided by each participant.

### Study Design

This cross-sectional survey was conducted from March 3-15, 2020. During this period, the total number of confirmed cases of COVID-19 exceeded 80,000 in China [36]. Chinese college students were invited to participate in a web-based survey though the Wenjuanxing platform. In total, 1676 college students participated in this study.

### Measurement of Panic

Panic was measured using the Phobia subscale of the self-reported Symptom Checklist 90 (SCL-90) [37]. This scale consists of 7 items that mainly cover phobia symptoms, with an emphasis on situations with limited availability of help and avoidance behavior. The score for each item ranges from 0-4. If a score is >2 (above average), some phobia symptoms may be present [38]. The Cronbach α coefficient of the total scale was .925.

### Measurement of Health Anxiety

The Health Anxiety Inventory (HAI) was used to measure health anxiety [39]. The HAI is an 18-item scale, and each item has 4 answer options ranging from 0 (“I do not”) to 3 (“I spend most of my time”) [39]. The presence of health anxiety is defined as a total score ≥15 [40]. The Cronbach α coefficient of the total scale was .818.

### Measurement of Health Risk Communication of COVID-19

Health risk communication of COVID-19 was measured through two aspects: COVID-19–related perceptions and knowledge of preventive behaviors.

The COVID-19–related perceptions included knowledge of the prognosis of COVID-19, understanding of the risk of COVID-19, perceived lethality of COVID-19, perceived severity of COVID-19, feeling that it is difficult to protect oneself from being affected by the pandemic, and being affected by the global spread. Each item had 5 answer options: very low, low, moderate, high, and very high. The responses were subsequently categorized as moderate (moderate/low/very low) or high (high/very high).

Knowledge of preventive behaviors included knowledge of preventive measures, awareness of handwashing, awareness of wearing a face mask, and impact of home quarantine on social contacts. The questions regarding preventive measures had 5 response options: very low, low, moderate, high, and very high. The responses were subsequently categorized as moderate (moderate/low/very low) or high (high/very high). The questions about handwashing, wearing a face mask, and the impact of home quarantine on social contacts had 5 response options: strongly disagree, slightly disagree, moderate, slightly agree, and strongly agree. The responses were subsequently categorized as no (strongly disagree/slightly disagree/moderate) or yes (slightly agree/strongly agree).

### Measurement of Social Media Sources of COVID-19 Information

Social media use was measured by asking which sources the participant used to obtain COVID-19–related information during the last month. These sources included WeChat, Sina Weibo, websites, television, newspapers, broadcast, government notices, or family members or friends. Trusted information sources were identified by asking “What do you think is the most trusted source of information regarding COVID-19?” The answers were divided into two groups: mainstream media and nonmainstream media.

### Demographic Characteristics

The demographic factors examined included gender, major, monthly disposable income, region, number of cases in the participant’s province, and year of study. Gender was divided into male and female. Major was divided into medicine-related major and other. Year was categorized as Year 1/2 and Year 3/4/5. Monthly disposable income was categorized into three groups: ≤1000 yuan (≤US $151), 1001-2000 yuan (US $162-303), and >2000 yuan (>US $303). Region was divided into rural and urban. Confirmed cases per province was divided into <1000 and ≥1000.

### Statistical Analysis

First, comparisons between participants with and without panic or health anxiety were performed using chi-square tests for categorical variables. Next, a multivariable logistic regression analysis was conducted to identify which determinants contributed most to the likelihood of panic or health anxiety. Logistic regression analyses were used to explain the associations between the prevalence of panic, health anxiety, social media, and health risk communication after controlling for covariates. The aforementioned variables were all entered into the multivariate model. The Nagelkerke *R^2^* value was used as the coefficient of determination. Missing values were replaced with mean values. The Hosmer-Lemeshow test was used to examine the goodness-of-fit of the model, and a *P* value >.05 indicated acceptable fitness. Statistical analysis was conducted using SPSS 21.0 (IBM Corp), and a two-tailed *P* value <.05 was viewed as statistically significant.

## Results

### Participant Characteristics

Among the 1676 students, the prevalence of panic was 17.2% (n=288), and the prevalence of health anxiety was 24.3% (n=408). The mean age of the students was 20.17 years (SD 1.497). As shown in Tables 1-3, most of the college students were female (1088/1676, 64.9%). Most of the participants (1220/1676, 72.8%) were in Year 1 or Year 2. In total, 1235/1676 students (73.7%) were from urban areas. According to our results, only 121/1676 students (7.2%) were from Hubei, Guangdong, Henan, Zhejiang, or Hunan Province, each of which had more than 1000 confirmed cases of COVID-19.

**Table 1:** Demographic factors of the respondents and differences between panic and health anxiety (N=1676).

Demographic factor			Total, n (%)		Panic							Health anxiety						
				Yes, n (%)		No, n (%)	*χ^2^* ^a^		*P* value		Yes, n (%)		No, n (%)		*χ^2^* ^a^		*P* value	
**Gender**								22.499		<.001						0.116		.73
	Male	588 (35.1)		136 (8.1)		452 (27.0)					146 (8.7)		442 (26.4)					
	Female	1088 (64.9)		152 (9.1)		936 (55.8)					262 (15.6)		826 (49.3)					
	Total	1676 (100)		288 (17.2)		1388 (82.8)					408 (24.3)		1268 (75.7)					
**Major**								5.735		.02						0.123		.73
	Medicine-related major	1219 (72.7)		193 (11.5)		1026 (61.2)					294 (17.5)		925 (55.2)					
	Other	457 (27.3)		95 (5.7)		362 (21.6)					114 (6.8)		343 (20.5)					
	Total	1676 (100)		288 (17.2)		1388 (82.8)					408 (24.3)		1268 (75.7)					
**Year of study**								4.539		.03						16.743		<.001
	1/2	1220 (72.8)		195 (11.6)		1025 (61.2)					265 (15.8)		955 (57.0)					
	3/4/5	456 (27.2)		93 (5.5)		363 (21.7)					143 (8.5)		313 (18.7)					
	Total	1676 (100)		288 (17.2)		1388 (82.8)					408 (24.3)		1268 (75.7)					
**Monthly disposable income (yuan)^b^**								1.961		.38						4.479		.11
	≤1000	366 (21.8)		67 (4.0)		299 (17.8)					103 (6.1)		263 (15.7)					
	1001-2000	1038 (61.9)		182 (10.9)		856 (51.2)					236 (14.1)		802 (47.9)					
	>2000	272 (16.2)		39 (2.3)		233 (13.9)					69 (4.1)		203 (12.1)					
	Total	1676 (100)		288 (17.2)		1388 (82.8)					408 (24.3)		1268 (75.7)					
**Region**								3.229		.07						0.188		.66
	Rural	441 (26.3)		88 (5.2)		353 (21.1)					104 (6.2)		337 (20.1)					
	Urban	1235 (73.7)		200 (11.9)		1035 (61.8)					304 (18.1)		931 (55.5)					
	Total	1676 (100)		288 (17.2)		1388 (82.8)					408 (24.3)		1268 (75.7)					
**Confirmed cases in province**								2.1		.15						4.406		.04
	<1000	1555 (92.8)		273 (16.3)		1282 (76.5)					369 (22.0)		1186 (70.8)					
	≥1000	121 (7.2)		1 (0.1)		106 (6.3)					39 (2.3)		82 (4.9)					
	Total	1676 (100)		288 (17.2)		1388 (82.8)					408 (24.3)		1268 (75.7)					

^a^All degrees of freedom are 1 except for monthly disposable income, for which the degrees of freedom are 2.

^b^1 yuan=US $0.14 on March 3, 2020.

**Table 2:** Knowledge of COVID-19–related risks and protective measures reported by the respondents and differences between panic and health anxiety (N=1676).

Health risk knowledge			Total, n (%)	Panic					Health anxiety							
				Yes, n (%)	No, n (%)		*χ^2^*_1_	*P* value		Yes, n (%)		No, n (%)		*χ^2^*_1_		*P* value
**Perception of COVID-19–related information**																
	**Knowledge of prognosis**					0.009		.92					15.479		<.001	
		Moderate	514 (30.7)	89 (5.3)	425 (25.4)					157 (9.4)		357 (21.3)				
		High	1162 (69.3)	199 (11.9)	963 (57.5)					251 (15.0)		911 (54.4)				
		Total	1676 (100)	288 (17.2)	1388 (82.8)					408 (24.3)		1268 (75.7)				
	**Understanding of the risk**					36.618		<.001					3.601		.06	
		Moderate	782 (46.7)	181 (10.8)	601 (35.9)					207 (12.4)		575 (34.3)				
		High	894 (53.3)	107 (6.3)	787 (47.0)					201 (12.0)		693 (41.3)				
		Total	1676 (100)	288 (17.2)	1388 (82.8)					408 (24.3)		1268 (75.7)				
	**Perceived lethality**					0.004		.95					49.315		<.001	
		Moderate	1300 (77.6)	223 (13.3)	1077 (64.3)					265 (15.8)		1035 (61.8)				
		High	376 (22.4)	65 (3.9)	311 (18.6)					143 (8.5)		233 (13.9)				
		Total	1676 (100)	288 (17.2)	1388 (82.8)					408 (24.3)		1268 (75.7)				
	**Feeling that it is difficult to protect oneself from the pandemic**					4.658		.03					22.688		<.001	
		Moderate	1482 (88.4)	244 (14.6)	1238 (73.9)					334 (19.9)		1148 (68.5)				
		High	194 (11.6)	44 (2.6)	150 (8.9)					74 (4.4)		120 (7.2)				
		Total	1676 (100)	288 (17.2)	1388 (82.8)					408 (24.3)		1268 (75.7)				
	**Perceived severity**					3.443		.06					21.551		<.001	
		Moderate	1228 (73.3)	225 (13.4)	1011 (60.3)					265 (15.8)		971 (57.9)				
		High	448 (26.7)	63 (3.8)	377 (22.5)					143 (8.5)		297 (17.7)				
		Total	1676 (100)	288 (17.2)	1388 (82.8)					408 (24.3)		1268 (75.7)				
	**Affected by global spread**					2.579		.11					24.624		<.001	
		Moderate	399 (23.8)	58 (3.5)	341 (20.3)					60 (3.6)		339 (20.2)				
		High	1277 (76.2)	230 (13.7)	1047 (62.5)					348 (20.8)		929 (55.4)				
		Total	1676 (100)	288 (17.2)	1388 (82.8)					408 (24.3)		1268 (75.7)				
**Knowledge of preventive behaviors**																
	**Knowledge of preventive measures**					3.566		.06					17.235		<.001	
		Moderate	43 (2.6)	12 (0.7)	31 (1.8)					22 (1.3)		21 (1.3)				
		High	1633 (97.4)	276 (16.5)	1357 (81.0)					386 (23.0)		1247 (74.4)				
		Total	1676 (100)	288 (17.2)	1388 (82.8)					408 (24.3)		1268 (75.7)				
	**Awareness of handwashing**					10.416		.001					1.269		.26	
		No	70 (4.2)	22 (1.3)	48 (2.9)					21 (1.3)		49 (2.9)				
		Yes	1606 (95.8)	266 (15.9)	1340 (80.0)					387 (23.1)		1219 (72.7)				
		Total	1676 (100)	288 (17.2)	1388 (82.8)					408 (24.3)		1268 (75.7)				
	**Awareness of wearing face masks**					4.452		.04					8.025		.005	
		No	45 (2.7)	13 (0.8)	32 (1.9)					19 (1.1)		26 (1.6)				
		Yes	1631 (95.8)	275 (16.4)	1356 (80.9)					389 (23.2)		1242 (74.1)				
		Total	1676 (100)	288 (17.2)	1388 (82.8)					408 (24.3)		1268 (75.7)				
	**Impact of home quarantine on social contacts**					1.588		.21					18.277		<.001	
		No	1045 (62.4)	189 (11.8)	856 (51.1)					218 (13.0)		827 (49.3)				
		Yes	631 (37.6)	99 (5.9)	532 (31.7)					190 (11.3)		441 (26.3)				
		Total	1676 (100)	288 (17.2)	1388 (82.8)					408 (24.3)		1268 (75.7)				

**Table 3:** Number of social media information sources and trust in sources reported by the respondents and differences between panic and health anxiety (N=1676).

Social media source		Total, n (%)	Panic				Health anxiety			
			Yes, n (%)	No, n (%)	*χ^2^*_1_	*P* value	Yes, n (%)	No, n (%)	*χ^2^*_1_	*P* value
**Number of information sources**					1.082	.30			0.711	.40
	≤3	745 (44.5)	136 (8.1)	609 (36.3)			174 (104)	571 (34.1)		
	≥4	931 (55.5)	152 (9.0)	779 (46.5)			234 (14.0)	697 (41.6)		
	Total	1676 (100)	288 (17.2)	1388 (82.8)			408 (24.3)	1268 (75.7)		
**Trust in information**					6.641	.01			20.757	<.001
	Nonmainstream	301 (18)	67 (4.0)	234 (14.0)			104 (6.2)	197 (11.8)		
	Mainstream	1375 (82)	221 (13.2)	1154 (67.9)			304 (18.1)	1071 (63.9)		
	Total	1676 (100)	288 (17.2)	1388 (82.8)			408 (24.3)	1268 (75.7)		

### Relationships Among Demographic Factors, Health Risk Communication, Social Media, and Panic

With respect to demographic factors, the groups showed significant differences in the distributions of gender, major, and year of study in the univariate analyses according to chi-square tests. No significant differences were found for monthly disposable income, region, or cases per province between the two groups.

Students who understood the risks of COVID-19 and felt less affected by the outbreak had lower panic levels. With respect to preventive knowledge, students who were aware that handwashing and wearing face masks can prevent COVID-19 had less panic. Regarding social media, students who trusted mainstream media information had less panic.

### Relationships Among Demographic Factors, Social Media, Health Risk Communication, and Health Anxiety

With respect to demographic factors, the groups showed significant differences in the distributions of year of study and cases per province in the univariate analyses according to chi-square tests. No significant differences were found for gender, major, monthly disposable income, or region between the two groups.

Regarding COVID-19–related perception, students who had knowledge of the prognosis of the disease and felt less affected by the outbreak had lower health anxiety. Students who thought COVID-19 was lethal and severe and who were affected by the global spread had more health anxiety. In terms of preventive knowledge, students who had knowledge of preventive measures and were aware that wearing face masks could prevent COVID-19 had less health anxiety. Students who considered that their social contacts were affected by home quarantine had more health anxiety. Regarding social media, participants who believed mainstream information was more trustworthy had less health anxiety.

### Risk Factors for Panic

Multivariable logistic regression analysis was conducted to identify which determinants contributed most to the likelihood of developing panic. The results of the unadjusted model and a model adjusting for potentially confounding demographic factors are reported. The confounders included in the adjusted model were major, gender, and year. The Nagelkerke pseudo-*R^2^* values for the unadjusted and adjusted models were 0.048 and 0.074, respectively. The Hosmer-Lemeshow tests demonstrated adequate fitness for the unadjusted (*χ^2^*_4_=2.733, *P*=.60) and adjusted (*χ^2^*_8_=5.790, *P*=.67) models. The variables in the adjusted model explained 7.4% of the variance in panic. The main effects of the adjusted model were similar to the crude results except for the awareness of handwashing. As shown in the adjusted model, a better understanding of the risk of COVID-19 (odds ratio [OR] 0.480, 95% CI 0.367-0.627) was identified as a significant protective factor for panic. These data are shown in Table 4.

**Table 4:** Results of logistic regression of risk factors for panic.

Variable		Unadjusted model^a^		Adjusted model^b^	
		OR^c^ (95% CI)	*P* value	OR (95% CI)	*P* value
**COVID-19–related perception**					
	Understanding the risk of COVID-19 (high vs moderate)	0.476 (0.366-0.621)	<.001	0.480 (0.367-0.627)	<.001
	Felt it was difficult to protect oneself from the pandemic (high vs moderate)	1.279 (0.881-1.855)	.20	1.243 (0.853-1.812)	.26
**Knowledge of preventive behaviors**					
	Awareness of handwashing (yes vs no)	0.533 (0.292-0.975)	.04	0.619 (0.336-1.143)	.13
	Awareness of wearing face masks (yes vs no)	0.892 (0.412-1.942)	.77	0.933 (0.427-2.034)	.86
**Information source**					
	Trusted information (mainstream media vs nonmainstream media)	0.738 (0.538-1.013)	.06	0.792 (0.574-1.092)	.15
**Covariates**					
	Gender (female vs male)	N/A^d^	N/A	0.526 (0.403-0.687)	<.001
	Year of study (3/4/5 vs 1/2)	N/A	N/A	1.268 (0.951-1.692)	.11
	Major (medicine-related major vs other)	N/A	N/A	0.720 (0.538-0.962)	.03

^a^Nagelkerke *R^2^*=0.048.

^b^Nagelkerke *R^2^*=0.074.

^c^OR: odds ratio.

^d^N/A: not applicable.

### Risk Factors for Health Anxiety

The results of the unadjusted model and a model adjusting for potentially confounding demographic factors are reported in Table 5. The confounders included in the adjusted model were cases per province and year of study. The Nagelkerke pseudo-*R^2^* values for the unadjusted and adjusted models were 0.110 and 0.119, respectively. The Hosmer-Lemeshow tests demonstrated adequate fitness for the unadjusted (*χ^2^*_8_=3.610, *P*=.89) and adjusted (*χ^2^*_7_= 3.080, *P*=.88) models. The variables in the adjusted model explained 11.9% of the variance in health anxiety. The main effects of the adjusted model were similar to the crude results except for perceived severity. As shown in the adjusted model, knowledge of prognosis (OR 0.708, 95% CI 0.551-0.910), knowledge of preventive measures (OR 0.380, 95% CI 0.195-0.742), awareness of wearing a face mask (OR 0.445, 95% CI 0.230-0.862), and trust in mainstream media (OR 0.613; 95% CI 0.461-0.816) were shown to be protective factors in predicting health anxiety. Perceived lethality (OR 1.860, 95% CI 1.408-2.459), being affected by the global spread (OR 1.936, 95% CI 1.405-2.669), and impact on social contacts (OR 1.420, 95% CI 1.118-1.802) were significant risk factors for health anxiety.

**Table 5:** Results of logistic regression of risk factors for health anxiety.

Variable		Unadjusted model^a^		Adjusted model^b^	
		OR^c^ (95% CI)	*P* value	OR (95% CI)	*P* value
**COVID-19–related perception**					
	Knowledge of prognosis (high vs moderate)	0.698 (0.544-0.896)	.005	0.708 (0.551,0.910)	.007
	Perceived lethality (high vs moderate)	1.898 (1.438-2.506)	<.001	1.860 (1.408,2.459)	<.001
	Felt it was difficult to protect oneself from the pandemic (high vs moderate)	1.231 (0.866-1.750)	.25	1.232 (0.865-1.753)	.25
	Perceived severity (high vs moderate)	1.309 (1.003-1.708)	.047	1.302 (0.996-1.701)	.05
	Affected by global spread (high vs moderate)	2.014 (1.464-2.772)	<.001	1.936 (1.405-2.669)	<.001
**Knowledge of preventive behaviors**					
	Knowledge of preventive measures (high vs moderate)	0.374 (0.193-0.728)	.004	0.380 (0.195-0.742)	.005
	Awareness of wearing face masks (yes vs no)	0.439 (0.228-0.844)	.014	0.445 (0.230-0.862)	.02
	Impact of home quarantine on social contacts (yes vs no)	1.434 (1.131-1.818)	.003	1.420 (1.118-1.802)	.004
**Social media**					
	Trusted information (mainstream media vs nonmainstream media)	0.611 (0.460-0.812)	0.001	0.613 (0.461-0.816)	.001
**Covariates**					
	Number of confirmed cases in province (≥1000 vs <1000)	N/A^d^	N/A	1.405 (0.927-2.219)	.11
	Year of study (3/4/5 vs 1/2)	N/A	N/A	1.453 (1.129-1.870)	.004

^a^Nagelkerke *R^2^*=0.110.

^b^Nagelkerke *R^2^*=0.119.

^c^OR: odds ratio.

^d^N/A: not applicable.

## Discussion

### Principal Findings

This study investigated the prevalence of panic and health anxiety among college students and explored the associations of health risk communication and social media with panic and health anxiety during the pandemic outbreak. Our results indicated that the prevalence of panic was 17.2% (288/1676) and the prevalence of health anxiety was 24.3% (408/1676). Previous studies have also reported that pandemics can trigger psychological symptoms [31,41,42]. If a pandemic constitutes an uncertain and threatening situation, it is more likely to trigger psychological symptoms. During pandemics, the number of people whose mental health is affected is greater than the number of people infected with the disease [43]. It is necessary to implement psychological interventions for college students during the COVID-19 pandemic.

Health risk communication was found to be important in predicting psychological symptoms among college students. With respect to risk perception, understanding the risk of COVID-19 was the only influencing factor for panic. Understanding the risk of COVID-19 could help relieve panic among college students. Similarly, knowledge of the prognosis of COVID-19 was a protective factor in predicting health anxiety. Individuals behave in a more reticent and conservative manner when they feel threatened by disease [44]. Awareness of risk may help students take effective measures to prevent infection and avoid panic and anxiety. Understanding the risk, prognosis, and routes of COVID-19 infection further decreases panic and health anxiety. Our results are in line with previous studies. Receiving more health information is correlated with lower levels of psychological distress [45]. Properly understanding information is important for reducing negative psychological responses brought on by inaccurate perceptions [46]. Clear communication involving regular, accurate updates on the COVID-19 outbreak plays a critical role in developing psychological health [6]. At present, there are still limited effective treatments and vaccines for COVID-19, and the high infectivity, lethality, and global spread of the disease are causing health anxiety among college students.

Knowledge of preventive behaviors was another aspect of health risk communication that significantly predicted health anxiety among college students. Similarly, knowledge of preventive measures and of wearing a face mask were protective factors in predicting health anxiety. Accurate knowledge helped individuals react to and positively combat the outbreak, and it resulted in less negative emotion. Knowledge and guidance about preventive behaviors are important factors in mitigating the spread of COVID-19 [47] and allaying unrealistic or excessive psychological anxiety [48]. Almost 37.6% of students (631/1676) felt that their social contacts were impacted by the home quarantine measures. The loss of freedom and increase of boredom had marked effects. Limitation of social contacts has a series of negative effects on psychological health. The etiology of anxiety as an illness includes a number of interacting biological, psychological, and social factors [49]. Individuals with better social networks are less likely to report anxiety symptoms [50]. Using class-based social groups may be able to improve college students’ positive psychology and promote a positive atmosphere to enhance strong-tie relationships [51]. Even during the pandemic outbreak, social support plays a critical role in alleviating students’ negative psychological symptoms, including anxiety [14]. Students can keep in touch with their friends or relatives on the web or by telephone to maintain social connections.

Social media played a critical role in psychological health. Students who believed that mainstream information was more trustworthy experienced less anxiety. Social media was the primary means of distributing information. Based on our results, 55% of students obtained COVID-19–related information in more than 4 ways. Mass media exposure to “infomedia” through social media platforms can create anxiety because rumors, “fake news,” and conspiracy theories make it difficult to find trustworthy information [52]. Misinformation has caused anxiety and even hampered the response to the outbreak. Students spend a lot of time on the internet, and they are more likely to be misguided and experience triggering of anxiety symptoms. Appropriate guidance from authorities, meanwhile, can prevent individuals from overreacting to the disease and engaging in excessively avoidant behaviors [53]. Official public health organizations provide accurate information on measures to avoid COVID-19 [54], and the information is considered trustworthy and reliable [55]. During the pandemic, most people want to receive information from municipal health services, health care providers, and official media sources [56]. In the face of public health emergencies, accurate and authoritative information is important for relieving psychological symptoms among college students.

### Implications

There are several implications of this study for clinical practitioners and policy makers. First, more attention should be paid to the psychological health of college students during the COVID-19 pandemic, and protective measures must be increased. Regardless of whether they were infected, students experienced psychological impacts from the outbreak. The delayed college start, uncertainty, and potential negative impact on academic progression may enhance the psychological burden on college students. Second, social media should be held responsible for providing correct and evidence-based information. Our study indicated that 55.5% (931/1676) of students obtained COVID-19–related information from more than four sources. Social media reporting can have both positive and negative consequences, and it had a strong influence on the psychological health of students. Information on social media platforms should be managed to quickly reduce the spread of fear and uncertainty and enhance public trust in public health measures [52]. Third, social isolation should be avoided. Imposed quarantine, including separation from friends or relatives and a departure from usual daily routines, is an unpleasant experience [57]. Additionally, social isolation caused an increase in anxiety. The need for social support has increased during the current pandemic. It is necessary to communicate socially via the internet or by telephone during the COVID-19 pandemic. Finally, health risk communication is essential during the outbreak. Based on our research, almost all students possessed knowledge related to the prevention of COVID-19. While the severity of the pandemic can trigger psychological symptoms in college students, risk perception promotes appropriate practices among students [58]. Our results indicated that 95.8% of college students (1606/1676) believed that preventive behaviors were effective. These behaviors provided the students with a sense of security and decreased their fear and anxiety.

### Limitations

Several limitations should be considered in this study. First, conclusions on causality cannot be drawn due to the cross-sectional design. Second, given the use of a web-based survey, there may be some response bias. Third, the explained variance was low.

### Conclusion

Psychological symptoms among college students were found to be at high levels during the COVID-19 pandemic. Understanding the risk of COVID-19 was a protective factor for panic. Trust in mainstream media, knowledge of preventive measures of COVID-19, and knowledge of its prognosis were protective factors for anxiety. However, perceived lethality, the global spread, and impact on social contacts were risk factors for health anxiety. Effective health risk communication and scientific and evidenced-based information should be reported through social media. The psychological health of college students should be considered. Future research should focus on intervention measures to ensure college students’ psychological well-being during a global pandemic outbreak.

## Abbreviations

HAI: Health Anxiety Inventory
OR: odds ratio
SCL-90: Symptom Checklist 90
WHO: World Health Organization
